# Supplementary material for: Pregnancy duration and breast cancer risk
Source: Nat Commun. 2018 Oct 23;9:4255. doi: 10.1038/s41467-018-06748-3 (PMC6199327; doi:10.1038/s41467-018-06748-3)

Supplementary Information

Pregnancy duration and breast cancer risk

Husby et al.

Supplementary Tables

Supplementary Table 1. Breast cancer events and person-years according to the duration of 1st to 5th pregnancy in the Danish cohort[a].

Pregnancy duration[b]	1st pregnancy		2nd pregnancy		3rd pregnancy		4th pregnancy		5th pregnancy	
	Breast cancer events	Person-years in 1000s	Breast cancer events	Person-years in 1000s	Breast cancer events	Person-years in 1000s	Breast cancer events	Person-years in 1000s	Breast cancer events	Person-years in 1000s
No *n*th pregnancy	8,028	23,369.9	18,551	27,787.9	46,597	39,725.9	58,065	44,614.3	60,732	45,788.8
20-27	31	12.0	18	8.4	8	3.6	4	1.3	*	0.3
28-29	32	15.1	20	10.5	14	4.7	*	1.3	*	0.5
30	22	12.0	14	8.4	13	3.7	*	1.0	*	0.3
31	24	13.7	19	9.4	13	3.6	*	1.3	*	0.4
32	42	21.1	34	14.5	14	6.3	*	1.9	*	0.6
33	52	26.2	44	18.9	15	8.0	6	2.3	*	0.7
34	78	42.3	61	30.8	25	12.3	*	3.4	*	0.9
35	138	64.1	90	46.6	34	18.1	13	5.4	*	1.3
36	241	125.2	210	96.6	76	38.8	22	10.5	7	2.9
37	437	228.2	417	204.4	170	83.0	53	23.0	14	5.5
38	912	495.1	1,044	524.9	484	213.9	113	50.3	22	12.2
39	1,818	944.2	2,057	1,067.0	857	392.7	210	89.8	25	18.9
40	3,809	2,046.2	4,521	2,241.7	1,623	776.8	351	171.3	69	37.1
41	1,906	1,017.6	2,006	1,057.3	722	359.1	169	74.1	21	14.7
≥42	990	560.4	18,551	480.7	324	167.5	61	34.2	13	6.9
Missing duration[c]	896	450.7	894	470.4	433	173.3	61	41.3	18	10.0
Civil register childbirths[d]	41,892	16,684.2	30,325	12,049.8	9,926	4,137.0	2,187	1,001.5	423	226.4

* Cells with three events or less.

[a] All events and person-years from 10 years after latest childbirth.

[b] Pregnancies registered to have lasted less than 20 weeks or more than 45 gestational weeks was also included in the analysis, but constituted combined only <0.01 % of observation time in Denmark.

[c] Childbirths registered in the Birth Registers, but with missing duration of pregnancy.

[d] Childbirths registered in the civil registration system, but not in the Birth Register. Predominantly childbirths before January 1, 1978. After this date only 3.34% of childbirths are not reported in the Medical Births Register.

Supplementary Table 2. Breast cancer events and person-years according to the duration of 1st to 5th pregnancy in the Norwegian cohort[a].

	1st pregnancy		2nd pregnancy		3rd pregnancy		4th pregnancy		5th pregnancy	
**Pregnancy duration**[b]	Breast cancer events	Person-years in 1000s	Breast cancer events	Person-years in 1000s	Breast cancer events	Person-years in 1000s	Breast cancer events	Person-years in 1000s	Breast cancer events	Person-years in 1000s
No *n*th pregnancy	2,880	13,860.7	5,876	15,710.2	14,458	20,978.2	19,097	23,943.2	20,410	24,843.7
20-27	25	19.6	22	13.3	10	7.4	7	2.3	*	0.8
28-29	28	15.6	16	10.3	7	5.1	*	1.7	*	0.7
30	12	12.7	20	8.2	9	4.1	*	1.2	*	0.5
31	30	16.3	24	11.4	9	5.3	*	1.8	*	0.6
32	41	21.7	25	15.6	9	7.0	*	2.6	*	0.9
33	50	32.5	40	21.1	18	10.9	5	3.5	*	0.9
34	75	51.0	45	35.4	30	17.6	9	6.1	*	2.1
35	117	83.1	96	60.9	47	29.6	16	11.1	*	3.5
36	232	137.3	172	105.9	81	53.2	24	17.8	6	5.4
37	365	244.1	315	210.4	188	108.7	43	37.6	14	11.8
38	783	515.9	879	537.8	401	271.9	132	91.0	27	25.4
39	1,810	1,149.8	2,016	1,294.8	1,021	628.2	271	194.3	61	55.9
40	2,677	1,744.5	2,953	1,882.2	1,343	880.2	381	260.5	82	74.5
41	2,180	1,477.9	2,088	1,402.0	983	649.0	252	185.2	52	52.5
≥42	1,516	1,093.1	1,310	879.7	582	408.2	166	121.0	37	33.2
Missing duration[c]	382	294.1	476	330.2	204	160.1	71	257.5	13	15.8
Civil register childbirths[d]	7,557	4,394.7	4,391	2,639.2	1,377	956.5	309	50.7	75	62.9

* Cells with three events or less.

[a] All events and person-years from 10 years after latest childbirth.

[b] Pregnancies registered to have lasted less than 20 weeks or more than 45 gestational weeks was also included in the analysis, but constituted combined only 0.13% of observation time in Norway.

[c] Childbirths registered in the Birth Registers, but with missing duration of pregnancy.

[d] Childbirths registered in the civil registration system, but not in the Birth Register. Predominantly childbirths before January 1, 1967. After this date only 3.85% of childbirths are not reported in the Medical Births Register.

Supplementary Figures

Supplementary Figure 1. Long-term relative risk of breast cancer after first(■), second(■), and third childbirth(■), compared with one childbirth less, by age at childbirth in Denmark. Horizontal lines show average effect of first(—), second(—), and third(—) childbirth in early age. Estimates are adjusted for educational attainment and employment position. Error bars indicate 95% confidence intervals.

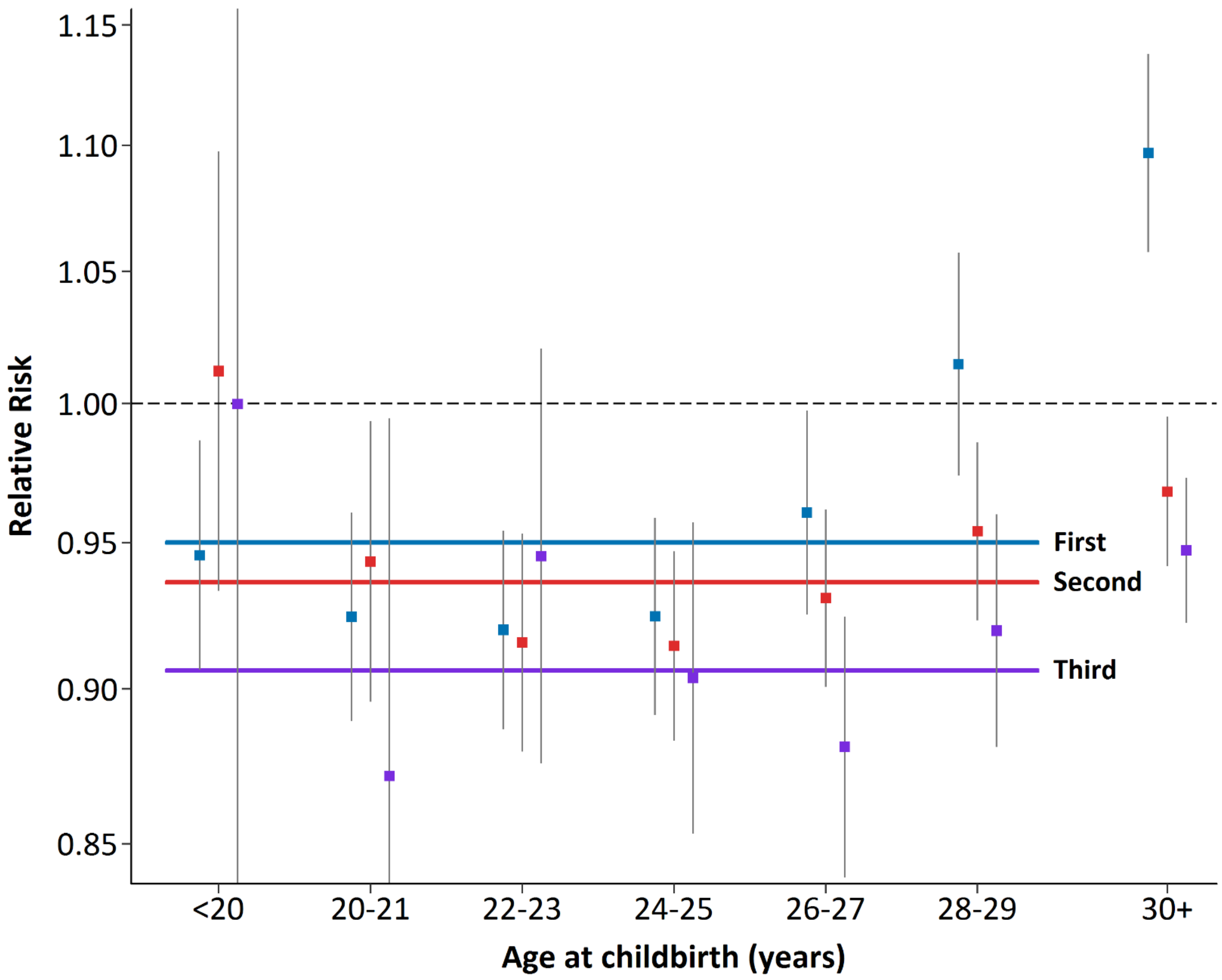

Supplementary Figure 2. Long-term relative risk of breast cancer after a second, third, or additional early age childbirth compared with one childbirth less, according to duration of pregnancy among women in Denmark and Norway. Error bars indicate 95% confidence intervals.

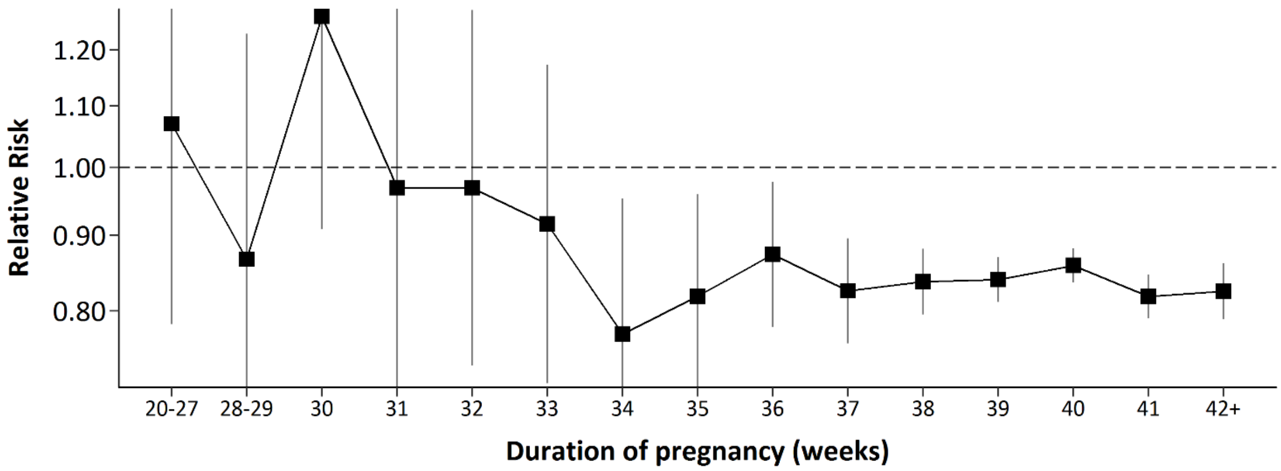

Supplementary Figure 3. Long-term relative risk of breast cancer after an early age childbirth according to duration of pregnancy, stratified on relative birthweight (small for gestational age (SGA) vs. non-SGA) in the Danish cohort. Error bars indicate 95% confidence intervals.

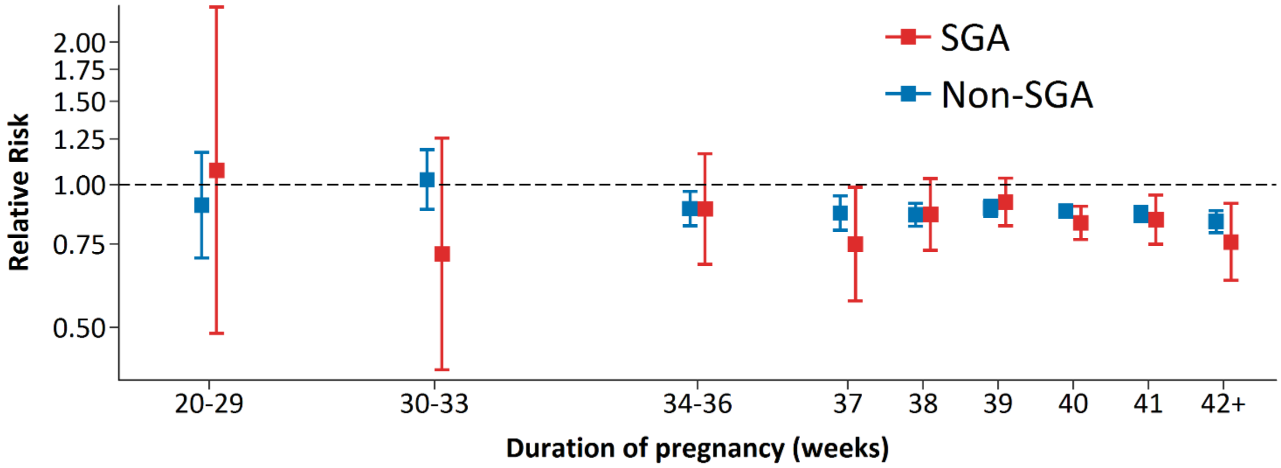

Supplementary Figure 4. Long-term relative risk of breast cancer after an early age pregnancy (abortions and childbirths) compared with one pregnancy less, according to duration of pregnancy in the Danish cohort. Error bars indicate 95% confidence intervals.

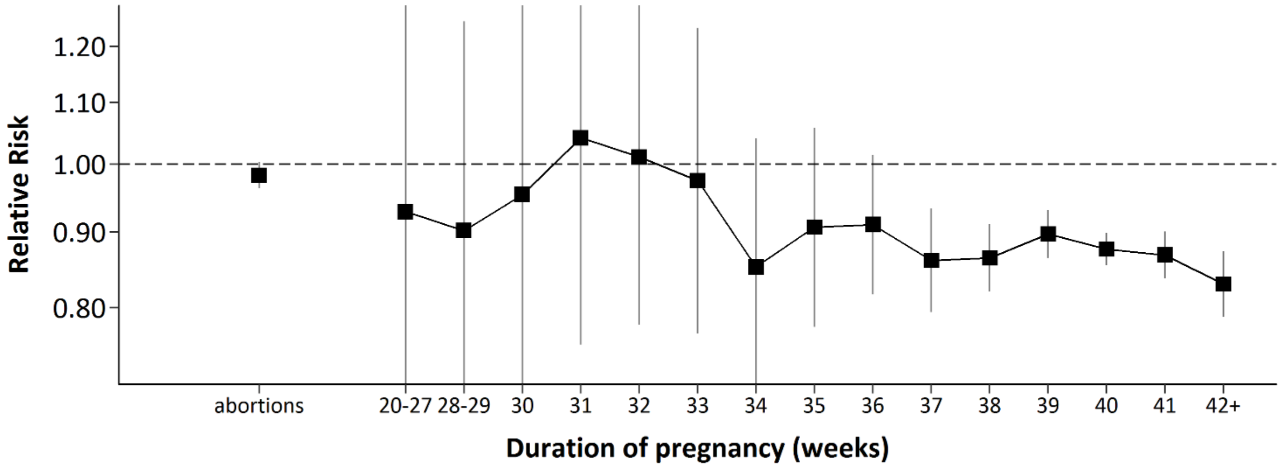

Supplementary Figure 5. Effect of different socioeconomic factors on long-term relative risk of breast cancer after childbirth in Denmark, by pregnancy duration.

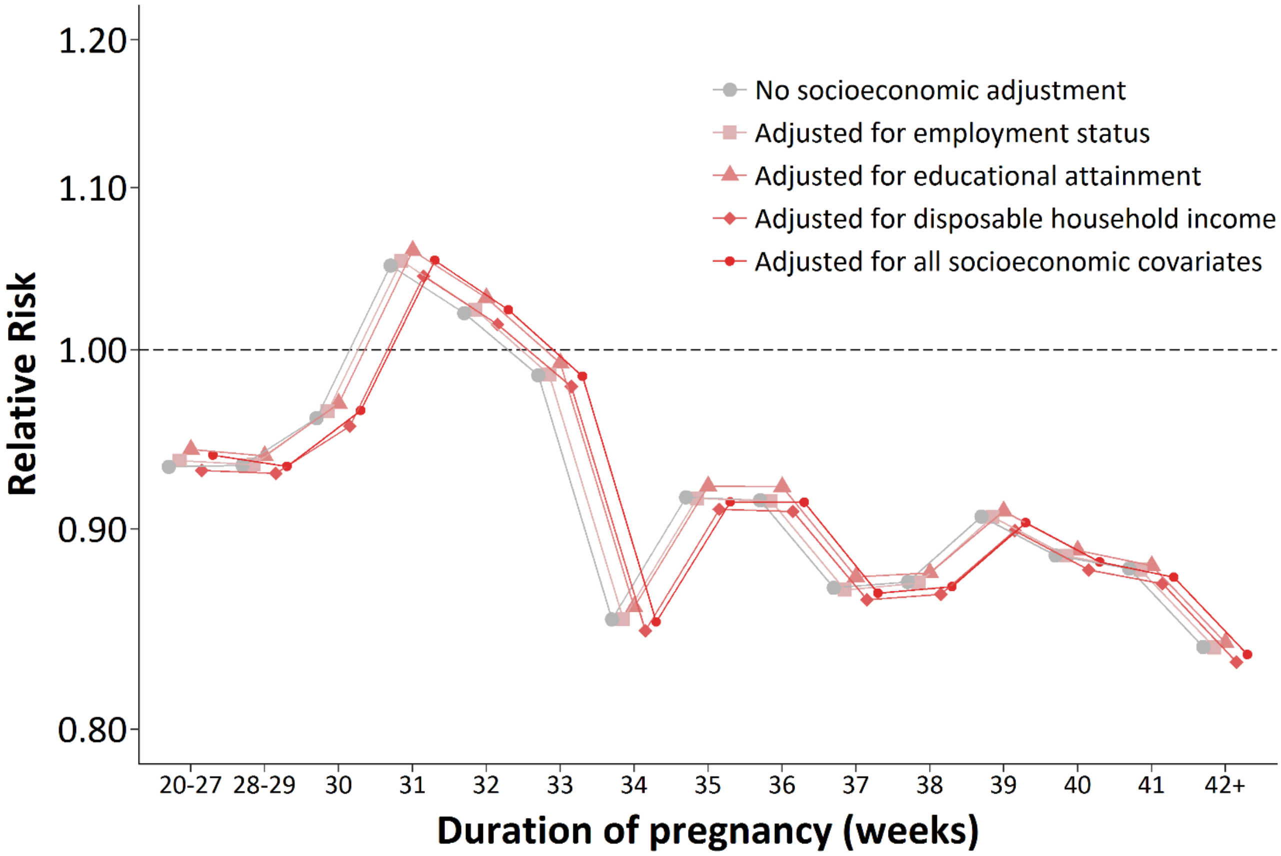

Supplementary Figure 6. Deviance between model in Figure 2C and three different threshold models according to threshold value. (A) Simple week-specific threshold model, (B) Threshold model which tolerated variation in the effect of parity and (C) Threshold model which tolerated variation in the effect of both parity and country.

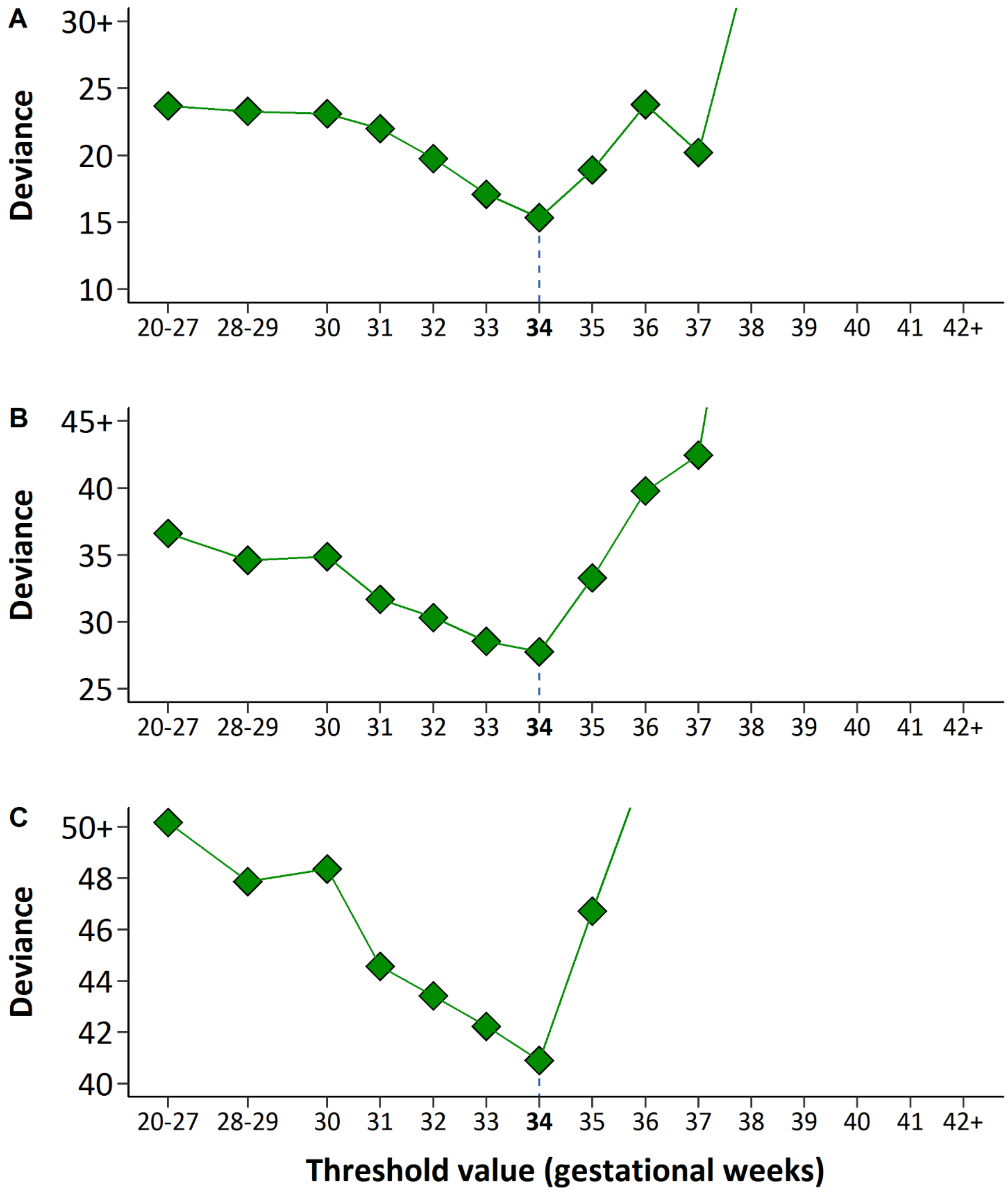

Supplement: Supplementary file 1 — Supplementary Information [file 41467_2018_6748_MOESM1_ESM.pdf]
